# Alcohol Consumption and Hepatitis C Virus (HCV) RNA Levels in HIV/HCV Coinfected Patients

**DOI:** 10.3390/v13050716

**Published:** 2021-04-21

**Authors:** Daniel Fuster, David Nunes, Debbie M. Cheng, Richard Saitz, Jeffrey H. Samet

**Affiliations:** 1 Department of Internal Medicine, Addiction Unit Hospital Universitari Germans Trias i Pujol, Department of Medicine, Universitat Autònoma de Barcelona, 08916 Badalona, Spain; 2 Section of Gastroenterology, Department of Medicine, Boston Medical Center and Boston University School of Medicine, Boston, MA 02118, USA; dnunes@bu.edu; 3 Clinical Addiction Research and Education (CARE) Unit, Section of General Internal Medicine, Department of Medicine, Boston Medical Center and Boston University School of Medicine, Boston, MA 02118, USA; dmcheng@bu.edu (D.M.C.); rsaitz@bu.edu (R.S.); Jsamet@bu.edu (J.H.S.); 4 Department of Biostatistics, Boston University School of Public Health, Boston, MA 02118, USA; 5 Department of Community Health Sciences, Boston University School of Public Health, Boston, MA 02118, USA; 6 Grayken Center on Addiction, Boston Medical Center, Boston, MA 02118, USA

**Keywords:** HIV, chronic HCV infection, HCV RNA levels, alcohol use disorder, unhealthy alcohol use, alcohol

## Abstract

Background: The impact of Hepatitis C virus (HCV) RNA levels on the evolution of chronic HCV infection-related liver damage is controversial. Heavy alcohol use is believed to have a deleterious impact on the course of HCV disease, but current knowledge about the possible effect of alcohol use on HCV RNA levels in HIV/HCV coinfected patients is limited. Methods: We examined 107 HIV/HCV-infected individuals with current or past unhealthy alcohol use to assess the association between alcohol consumption (any drinking vs. abstinent) and HCV RNA levels. Results: Participants were 75% male, with a mean age of 43 years, and 63% were on antiretroviral therapy. Mean (SD) log HIV RNA was 3.1 (1.4) and mean (SD) log HCV RNA was 6.1 (0.8). Past-month alcohol use was present in 38% of participants. In a multivariable linear regression analysis we found no significant differences in mean log HCV RNA levels between those reporting alcohol use and those who were abstinent [β (95%CI): −0.04 (−0.34, 0.26), *p* = 0.79)]. There was no significant association between any heavy drinking day and HCV RNA level (0.07, 95% CI: (−0.24, 0.38), *p* = 0.66). Conclusions: We did not detect significant associations between alcohol use and HCV RNA levels among HIV/HCV coinfected patients.

## 1. Introduction

Liver disease is a growing concern in HIV-infected people [1], as chronic hepatitis C virus (HCV) is frequent in HIV-infected people with past injection drug use [2]. The natural history of chronic HCV infection is negatively impacted by host-related factors, such as older age, male sex, other viral infections (e.g., Hepatitis B, HIV), polymorphisms of the IL28b gene, and coexistence of non-alcoholic fatty liver disease or diabetes, leading to a faster progression to end-stage liver disease [3,4]. The impact of viral-related factors (i.e., HCV RNA and HCV genotype) on the evolution of chronic HCV infection is controversial [5,6,7,8], but they are associated with HCV antiviral treatment response [9], particularly with interferon-based regimens.

Alcohol use is common in HCV-infected patients and correlates with liver injury and progression to end-stage liver disease [10,11]. Alcohol appears to promote HCV infection persistence, cytotoxicity, and oxidative stress [12], and has a negative impact on HCV antiviral treatment eligibility and adherence [13,14]. Some authors have described higher HCV RNA levels in people with HCV mono-infection and alcohol-use disorders [15,16] and yet a meta-analysis published in 2005, which included mostly studies of HCV mono-infected patients, failed to show an association between alcohol use and viral replication [17]. The case for alcohol having an impact on HCV RNA levels is also supported by in vitro evidence that alcohol metabolites increase the expression of HCV viral particles [18].

Additionally, alcohol use is twice as common in HIV-infected persons as in the general population [19,20]. Alcohol-use disorder has been associated with poor clinical outcomes, such as lower antiretroviral treatment (ART) initiation [21], lower engagement in proper HIV clinical care [22], treatment non-adherence [23], CD4 cell declines, and failure to suppress HIV replication [24,25].

In those with HIV/HCV coinfection, HCV RNA levels are higher than in HCV monoinfected patients [26], and tend to increase after the introduction of ART [27]. In coinfection, HCV RNA levels seem to be associated with the occurrence of chronic kidney disease [28] and one study correlated HCV RNA levels with mortality [29]. Thus, additional factors may be relevant in affecting HCV RNA levels in co-infected than in monoinfected people, and alcohol’s effect may differ in them too. Two prior studies have suggested that alcohol use (measured as a dichotomous variable through chart review or as a single question in an interview) might increase HCV RNA levels in coinfected people [30,31], but the effect of alcohol consumption on HCV RNA levels in coinfection is not fully understood.

Current standards of care of HCV antiviral treatment regimens with direct-acting antivirals (DAA) are far more effective than previously available interferon-containing regimens [32]. Alcohol use does not seem to have an impact on DAA treatment [33], but alcohol and other drugs were some of the main reasons why HCV treatment was not prescribed in the past [34], and abstinence from alcohol is still recommended by most HCV treatment guidelines [35]. Therefore, it is imperative for clinicians to understand the effects of certain comorbid conditions on the HCV-related liver disease process in HIV-infected patients with an effort to appropriately time treatment. The aim of the present study is to assess the association between alcohol use and HCV RNA levels in HIV/HCV coinfected patients with current or past unhealthy alcohol use.

## 2. Subjects and Methods

### 2.1. Study Design

This is an observational cohort study (the HIV Longitudinal Interrelationships of Viruses and Ethanol [HIV-LIVE] study) that included HIV-infected individuals with past or current unhealthy alcohol use, recruited in the Boston area between 2001 and 2003 and followed until 2006 [25].

Eligibility criteria for the HIV-LIVE study included: a documented HIV antibody test by enzyme-linked immunosorbent assay (ELISA) and confirmed by Western blot (medical record or tested at enrollment); affirmative responses to 2 or more CAGE alcohol screening questions [36] or physician investigator diagnosis of alcohol abuse or dependence; ability to speak English or Spanish; and at least 1 contact person to assist with follow-up [25].

Exclusion criteria included a score <21 on the 30-item Mini-Mental State Examination [37]; inability to provide informed consent or answer the interview questions; and plans to move from the Boston area in the subsequent 12 months [25].

Once enrolled, all participants underwent an interviewer-administered assessment. Interviewers collected demographic information and administered standardized questionnaires including a validated calendar method to verify alcohol consumption in the prior month [38].

All HIV-LIVE participants were evaluated for hepatitis C antibody at entry into the study. Those who were HCV-antibody-positive underwent HCV RNA assessment to verify the presence of HCV RNA. For the purpose of the present analysis, we included patients who had positive HCV antibody tests as well as a detectable HCV RNA, and an assessment of their alcohol consumption within 6 months of a measurement of detectable HCV RNA.

Therefore, those with spontaneously cleared HCV infection were not included.

Also, participants undergoing HCV antiviral therapy or those who had obtained a sustained viral response with prior HCV antiviral treatment were excluded.

### 2.2. Independent Variables:

Primary variables: Alcohol consumption was defined as consumption of any alcohol on any day during the past 30 days. Abstinence was no alcohol use in the past 30 days. Secondary measures of alcohol use were: any heavy drinking day (≥5 US standard drinks (12 oz of beer or 5 oz of wine) in any one day for men, and ≥4 drinks for women); total number of drinks in the past month, modeled as a continuous variable; and lifetime drinking history [39] in standard drinks, which was collected at study entry.

### 2.3. Primary Outcome

The primary outcome was log_10_ HCV RNA level. During the course of this study, the laboratory at Boston Medical Center changed its assay for hepatitis C RNA levels to measure international units instead of copies per milliliter. The appropriate formula for the conversion of copies/ml to international units was obtained from the laboratory and all HCV RNA results were converted to international units. Due to skewness in the distribution, HCV RNA was log_10_ transformed for analyses.

### 2.4. Covariates

The laboratory tests performed in the HIV-LIVE cohort determinations included CD4 cell count, aspartate aminotransferase (AST), and alanine aminotransferase (ALT).

Homelessness was defined as sleeping in a shelter or on the street for one or more nights in the last 6 months [40]. Use of antiretroviral therapy (ART) was defined in 3 categories: not on ART; on ART adherent; and on ART not adherent (<100% adherent during the prior 3 days) [41].

### 2.5. Statistical Analyses

Descriptive statistics were generated for all variables. Frequencies and proportions were obtained for categorical variables and means, standard deviations, medians, and ranges were obtained for continuous variables. Pearson correlation coefficients were obtained for each pair of independent variables and covariates; no pair of variables included in the analyses had r > 0.40. The associations between each alcohol-use variable and log HCV RNA were assessed using separate multiple linear regression models. Covariates included in each of the adjusted models were sex, age, race, homelessness, ART use, ART adherence, CD4 cell count, AST, and ALT. Covariates were not selected using statistical methods but rather based on clinical knowledge and the literature [40,42,43].

A secondary sensitivity analysis was performed including only the 69 participants who had alcohol consumption assessed within 30 days of their HCV RNA measurement. An additional analysis included the 87 study participants that had their alcohol use assessment before their HCV RNA measurements.

In addition, we also performed another sensitivity analysis using lifetime drinking history as the exposure measure. All analyses were conducted using two-sided tests defining a *p* < 0.05 as significant. Analyses were performed using SAS software (version 9.2; SAS Institute, Cary, NC, USA).

## 3. Results

**Figure 1:**
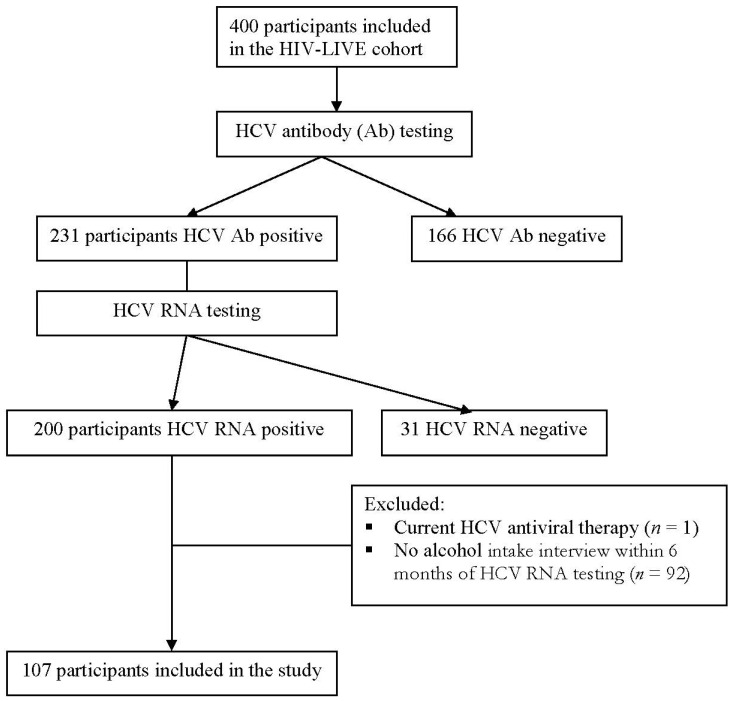
Flow diagram of enrollment into the study.

Among the 400 HIV-LIVE participants, 397 had available HCV antibody data; 231 were HCV-antibody-positive; and 166 were HCV-antibody-negative. Of the 231 HCV-antibody-positive subjects, 200 (86.5%) persons had detectable HCV RNA by PCR. One participant undergoing active interferon treatment was excluded, 17 had had prior treatment with interferon but did not respond to treatment. Of the subjects who were HCV RNA positive, 107 subjects had their alcohol intake interview within 6 months of their HCV RNA measurement and comprised the study sample for the current analyses (see Figure 1).

There were no significant differences in age, sex, race, homelessness, CD4 count, alcohol consumption, HIV, or HCV RNA between the 107 subjects that were included in the study and the 93 that were not (data not shown).

Characteristics of the study group are listed in Table 1. Participants were predominantly male (75%) with a diverse racial background. Mean age was 43 years; 63% were on ART, mean CD4 count was 404.9 cells/µL, and mean log HIV RNA was 3.1. Mean HCV RNA (standard deviation (SD) was 6.1 (0.8). Heavy alcohol consumption (for men ≤65 years of age >14 drinks per week or >4 drinks on any one occasion, and for men > 65 years and all women >7 per week or >3 on one occasion) was present in 31% of the participants, 8% reported lower risk amounts, and 59.8% reported abstinence in the prior 30 days. The median number of alcoholic drinks consumed in the past month among those reporting no use, lower-risk use, and heavy consumption were 0, 3, and 58, respectively. The median and interquartile range for lifetime drinking history was 1242.30 standard drinks (420-3384).

Table 1 also shows how participant characteristics are distributed according to drinking category [i.e., any use versus abstinence]. Individuals who reported any use were less likely to be on ART; if prescribed, they were less likely to be adherent with ART, and had higher mean log HIV RNA levels. There was no significant difference in the unadjusted mean log HCV RNA when examined by the main alcohol-use variable (any use versus abstinence) (*p* = 0.73).

The primary analysis did not detect a significant association between alcohol consumption and log HCV RNA (adjusted mean difference in log10 HCV RNA levels −0.04, 95% CI: (−0.34, 0.26), *p* = 0.79), controlling for age, sex, race, homelessness, CD4 count, and ART use and adherence. Similarly, there were no significant associations for any heavy drinking days or number of drinks per month, and the outcome log10 HCV RNA levels (0.07, 95% CI: (−0.24, 0.38), *p* = 0.66 for any vs. no heavy drinking days and 0.00 (−0.03, 0.02), *p* = 0.91 for every 30-drink increase per month, respectively) (Table 2).

In a secondary sensitivity analysis of those patients (*n* = 69) who had alcohol use measured within 30 days of their HCV measurement, the results were consistent with the primary analysis, and we found no significant associations between any of the three alcohol-use variables and HCV RNA (adjusted mean difference in log10 HCV RNA levels (95% CI)= 0.05 (−0.28, 0.38), *p* = 0.77; −0.02 (−0.36, 0.33), *p* = 0.91, and −0.01 (−0.03, 0.02), *p* = 0.67 for any alcohol use, any heavy drinking days, and for every 30-drink increase per month, respectively).

In a post hoc sensitivity analysis of patients (*n* = 87) who had alcohol use measured prior to their HCV RNA measurement, the results were also consistent with the primary analysis, adjusted mean difference in log10 HCV RNA levels (95% CI) = 0.07 (−0.27, 0.41), *p* = 0.69 for the unadjusted analysis and 0.08 (95% CI −0.26, 0.43) *p* = 0.63 for the adjusted analysis. We also performed an additional sensitivity analysis using lifetime drinking history as a measure of cumulative alcohol use (above or below the median). The results of this additional analysis were similar to the main analysis, with a beta estimate of 0.09 (95% confidence interval (CI) −0.21, 0.40), *p* = 0.55 for the unadjusted analysis and 0.13 (95% CI −0.16, 0.42) *p* = 0.38 for the adjusted analysis.

## 4. Discussion

In the present study, we did not detect a significant association between alcohol use and HCV RNA in a group of HIV/HCV coinfected individuals with current or past alcohol use. Secondary analyses requiring individuals to have had their HCV RNA measured within 30 days of their alcohol-use questionnaire also showed no significant association between any of our alcohol measurements and HCV RNA, nor were there associations with heavier alcohol use.

In this study, using more rigorous measures of alcohol consumption than those described in previous studies by Cooper and Fishbein [30,31], we failed to find an association between alcohol consumption and HCV RNA levels. Most participants in our study who drank, consumed heavy amounts and despite this, no association was detected. Cooper et al. used alcohol information extracted from clinical records, and observed that HCV RNA levels were found to have a sustained increase in those who drank more than 50 grams of alcohol per day [30]. Fishbein et al. studied HCV RNA in 264 people who injected drugs with and without HIV infection from NY and found that older age, HIV infection, and HCV genotype other than 1, were related to higher HCV RNA levels in the whole group [31]. Among those with HIV infection in that study (*n* = 142), detectable HIV RNA and alcohol use defined as a dichotomous variable derived from a single question from patient interviews regarding “any alcohol use in the 6 months prior to the observation point” were both predictors of increasing HCV RNA from baseline. The different and less detailed measures of alcohol use may account for the different results. In addition, different methods were employed to evaluate HCV RNA; we used baseline HCV RNA and both the Cooper et al. and Fishbein et al. papers measured evolution of HCV RNA levels between two time-points [30,31].

Our findings are not unexpected from the perspective that in HCV monoinfection, alcohol does not seem to have an impact on viral replication [17]. Given this and the current results, it is plausible to speculate that the negative effect that alcohol has in the progression of HCV-related liver injury is caused by mechanisms other than viral replication increasing HCV RNA levels. In this regard, the synergistic effects of HCV and alcohol use on cytotoxicity, oxidative stress, and immune response have been widely described in the literature [44,45,46].

This study’s strength is the validity of the alcohol questionnaire data, as the 30-day calendar review of alcohol intake and lifetime drinking history are more accurate than information extracted from medical records [30,31]. In addition, the prescription of HCV antiviral treatment in this cohort was very low, so the results are not likely to have been impacted by prescription and/or adherence to HCV antiviral treatment.

Limitations to the present study include having only HCV RNA measures at one time point, and not having alcohol assessment and HCV RNA determination within a 6-month time frame for all who had detectable HCV RNA. In addition, the power of the study was limited by the relatively small sample size. We note, however, that the observed point estimates were close to null. In post hoc power calculations, assuming the standard deviation of log HCV RNA for the abstinent and any alcohol use groups were 0.9 and 0.6 (as observed at baseline), respectively, this study would have approximately 80% power to detect a difference as small as 0.43 in mean log HCV RNA. Also, we cannot exclude that some patients with alcohol assessment after the determination of HCV RNA might have decreased their alcohol intake due to being diagnosed with chronic HCV infection.

It is also possible that our study population, which included patients with current or past unhealthy alcohol use, could include people who were abstinent who had already developed end-stage liver disease, biasing the study to the null.

In summary, despite examining several definitions of alcohol use, we did not find a significant association between alcohol consumption and HCV RNA levels in this exploratory analysis among HIV and HCV coinfected patients.

## Figures and Tables

**Table 1:** Characteristics of study participants–HIV/HCV coinfected persons with current or past unhealthy alcohol use.

Variable	Total (*n* = 107)	Abstinent (*n* = 64)	Any Use (*n* = 43)	*p*-Value
Age [Mean (SD)]	43.1 (7.5)	42.9 (7.5)	43.5 (7.3)	0.68
Sex, male [*n* (%)]	80 (75.0%)	47 (73.4%)	33 (76.7%)	0.70
Race [*n* (%)]				0.14
Black	40 (37.4%)	24 (37.5%)	16 (37.2%)	
White	40 (37.4%)	20 (31.2%)	20 (46.5%)	
Other	27 (25.2%)	20 (31.2%)	7 (16.3%)	
Homeless [*n* (%)]	32 (29.9%)	16 (25.0%)	16 (37.2%)	0.18
ART				<0.05
Adherent	52 (48.6%)	37 (57.8%)	15 (34.9%)	
Not adherent	15 (14.0%)	6 (9.4%)	9 (20.9%)	
No ART	40 (37.4%)	21 (32.8%)	19 (44.2%)	
Drinks per month				<0.01
Mean (SD)	58.3 (177.3)	0	145.1 (257.7)	
Median (IQR)	0 (0, 30)	0	38 (12, 120)	
Mean log HCV RNA (SD)	6.1 (0.8)	6.1 (0.9)	6.1 (0.6)	0.73
Mean log HIV RNA (SD)	3.1 (1.4)	2.8 (1.3)	3.2 (1.9)	0.03
Mean CD4 (SD) [cells/mm^3^]	404.9 (274.2)	421.7 (290.3)	381.1 (251.2)	0.46
Mean ALT (SD) [IU/L]	65.5 (51.0)	60.9 (50.3)	71.2 (52.8)	0.38
Mean AST (SD) [IU/L]	69.2 (48.4)	64.2 (48.1)	74.7(49.4)	0.28

SD: standard deviation, IQR: interquartile range. ART: highly active antiretroviral therapy.

**Table 2:** Multivariable analysis of the association between alcohol use and log HCV RNA in a cohort of HIV and HCV coinfected persons with current or past unhealthy alcohol use.

	Model I		Model II		Model III	
	β (95% CI)	*p*-Value	β (95% CI)	*p*-Value	β (95% CI)	*p*-Value
**Alcohol measure**						
Any use vs. abstinent	−0.04 (−0.34, 0.26)	0.79				
Any heavy drinking day			0.05 (−0.28, 0.38)	0.78		
Drinks/month *					0.00 (−0.02, 0.03)	0.91
**Covariates**						
Age	0.01 (−0.02, 0.03)	0.62	0.00 (−0.02, 0.03)	0.65	0.01 (−0.02, 0.03)	0.64
Female	−0.10 (−0.43, 0.24)	0.58	−0.09 (−0.43, 0.25)	0.60	−0.10 (−0.44, 0.24)	0.58
Black race	0.16 (−0.19, 0.50)	0.37	0.17 (−0.18, 0.51)	0.35	0.16 (−0.18, 0.51)	0.36
Homeless	0.15 (−0.17, 0.46)	0.36	0.14 (−0.17, 0.45)	0.37	0.14 (−0.17, 0.46)	0.37
CD4	0.00 (0.00, 0.00)	0.49	0.00 (0.00, 0.00)	0.50	0.00 (0.00, 0.00)	0.49
ART Adherent	0.09 (−0.25, 0.43)	0.61	0.11 (−0.24, 0.47)	0.53	0.10 (−0.24, 0.43)	0.57
ART Not adherent	0.19 (−0.26, 0.63)	0.41	0.18 (−0.27, 0.63)	0.43	0.18 (−0.26; 0.63)	0.42

β (95% CI) = Mean difference in log HCV RNA for exposed versus unexposed (95% confidence interval); * per 30 drinks per month increase.

## Data Availability

The raw data presented in this study are available to any scientist wishing to use them for non-commercial purposes on request from the corresponding author, without breaching participant confidentiality. The data are not publicly available due to privacy.
